# Thrombin Cleavage of Osteopontin Modulates Its Activities in Human Cells* In Vitro* and Mouse Experimental Autoimmune Encephalomyelitis* In Vivo*

**DOI:** 10.1155/2016/9345495

**Published:** 2016-07-13

**Authors:** Elena Boggio, Chiara Dianzani, Casimiro Luca Gigliotti, Maria Felicia Soluri, Nausicaa Clemente, Giuseppe Cappellano, Erika Toth, Davide Raineri, Benedetta Ferrara, Cristoforo Comi, Umberto Dianzani, Annalisa Chiocchetti

**Affiliations:** ^1^Department of Health Sciences and Interdisciplinary Research Center of Autoimmune Diseases (IRCAD), “A. Avogadro” University of Piemonte Orientale (UPO), 28100 Novara, Italy; ^2^Department of Drug Science and Technology, University of Torino, 10125 Torino, Italy; ^3^Biocenter, Division for Experimental Pathophysiology and Immunology, Laboratory of Autoimmunity, Medical University of Innsbruck, 6020 Innsbruck, Austria; ^4^Department of Translational Medicine, Neurology Unit, “A. Avogadro” UPO, 28100 Novara, Italy

## Abstract

Osteopontin is a proinflammatory cytokine and plays a pathogenetic role in multiple sclerosis and its animal model, experimental autoimmune encephalomyelitis (EAE), by recruiting autoreactive T cells into the central nervous system. Osteopontin functions are modulated by thrombin cleavage generating N- and C-terminal fragment, whose individual roles are only partly known. Published data are difficult to compare since they have been obtained with heterogeneous approaches. Interestingly, thrombin cleavage of osteopontin unmasks a cryptic domain of interaction with *α*_4_ *β*_1_ integrin that is the main adhesion molecule involved in lymphocyte transmigration to the brain and is the target for natalizumab, the most potent drug preventing relapses. We produced recombinant osteopontin and its N- and C-terminal fragments in an eukaryotic system in order to allow their posttranslational modifications. We investigated,* in vitro, *their effect on human cells and* in vivo* in EAE. We found that the osteopontin cleavage plays a key role in the function of this cytokine and that the two fragments exert distinct effects both* in vitro *and* in vivo*. These findings suggest that drugs targeting each fragment may be used to fine-tune the pathological effects of osteopontin in several diseases.

## 1. Introduction

Osteopontin (OPN) is a matricellular protein originally isolated from the bone, expressed by various cell types including macrophages, dendritic cells, and activated T cells. OPN mediates several biological functions such as bone remodeling, macrophage response, cell migration, and adhesion, and it is involved in the pathogenesis of several diseases including atherosclerosis, cancer, chronic inflammatory diseases, and several autoimmune diseases [1–3]. OPN costimulates T cell activation and supports differentiation of proinflammatory T helper 1 (Th1) and Th17 cells [4, 5].

OPN is a member of the SIBLING (Small Integrin Binding Ligand N-linked Glycoprotein) protein family. It has two calcium binding sites, two putative heparin binding domains, and multiple adhesion motifs which allow interaction with several receptors and cell types [6]. OPN biological functions are influenced by posttranslational modifications, such as phosphorylation, glycosylation, and protein cleavage mediated by thrombin and metalloproteinases [7–9]. OPN has RGD (arginine-glycine-aspartate) integrin binding domain, through which it mediates interactions with *α*v*β*1, *α*v*β*3, *α*v*β*5, *α*v*β*6, *α*8*β*1, and *α*5*β*1 integrins [10–13]. Moreover, *α*9*β*1, *α*4*β*1, and *α*4*β*7 integrins bind to a cryptic SVVYGLR (SLAYGLR in mice) sequence which is exposed upon thrombin cleavage occurring at the Arg168-Ser169 site near to the RGD motif [14–16]. This cleavage generates two fragments, the N- and C-terminal, displaying functional differences from the full length protein [17]. The N-terminal fragment (OPN-N, approx. 35 kDa) binds to integrins through the RGD or the cryptic binding site, promotes IFN-*γ* secretion in T cells, and stimulates cell migration by binding to *α*9*β*1 and *α*4*β*1 [18, 19]. The C-terminal fragment (OPN-C, approx. 25 kDa) inhibits IL-10 secretion and stimulates cell-cell adhesion by interacting with CD44 isoforms containing the v6 and v7 domains [20, 21].

OPN is highly expressed in several tumors, where it can be cleaved by thrombin and acts as a proangiogenic factor [22–25]. Moreover, the OPN:CD44 interaction promotes metastasis dissemination in a variety of malignancies [26]. The individual role of OPN-N and OPN-C has been investigated mainly in cancer cells because of the expression of both OPN and activated thrombin in the microenvironment of several tumors [22, 27, 28]. On the contrary, little information is available on their role in autoimmune diseases. Full length OPN (OPN-FL) is expressed at similar levels in the synovial fluid of patients with rheumatoid arthritis (RA) and in those with osteoarthritis, but OPN-N levels in RA synovial fluid samples are around 30-fold higher than in those from osteoarthritis and correlate with the disease status [29].

A considerable body of evidence suggests that OPN plays a detrimental role in multiple sclerosis (MS) and its animal model, experimental autoimmune encephalomyelitis (EAE) [30–32]. In MS lesions, high OPN levels are present in the perivascular cuff that surrounds inflamed blood vessels, contains inflammatory lymphocytes, and is delimited by the endothelium and the basement membrane. At this site, OPN plays a role in lymphocyte recruitment into the MS lesion, which involves *α*4*β*1 integrin that is the target of the anti-MS drug natalizumab, a humanized monoclonal antibody that has had benefic effects for relapses prevention in RR-MS [33]. OPN binds to *α*4*β*1 integrin only upon cleavage by thrombin which unmasks two *α*4*β*1 integrin binding sites located into the N-terminal fragment. Hur et al. showed that the administration of recombinant OPN-FL exacerbates EAE, but the relative role of OPN-N and OPN-C is not known [34]. However, thrombin-mediated cleavage may play a role, since thrombin activity increases with the progression of neuroinflammation, and it is detectable in the demyelinating lesions where also OPN is present at high levels [35, 36]. Moreover,* in vivo* administration of hirudin, a thrombin inhibitor, decreases clinical severity, demyelination, and secretion of Th1- and Th17-type cytokines in EAE [37, 38].

OPN modulates several cell activities* in vitro*, but the role of OPN cleavage and the relative role of OPN-C and OPN-N are only partly known. Moreover, several available data are difficult to compare because they have been obtained with heterogeneous approaches, such as antibody-mediated blockage of the OPN receptors, use of OPNs produced in bacteria and eukaryotic cells, which influence the posttranslational modifications involved in OPN function, or use of a mixture of OPN fragments obtained by* in vitro* treatment with thrombin.

The aim of our research was to recapitulate,* in vitro*, the OPN effects on human cells with a particular focus on processes involved in MS relapse, that is, T cell and monocyte activation, T cell apoptosis, lymphocytes, and endothelial cell migration and adhesion, by using recombinant forms of OPN-FL, OPN-N, and OPN-C produced in an eukaryotic system in order to ensure their posttranslation modifications.

Moreover, we investigated the activity of these OPNs and a point-mutated form of OPN resistant to thrombin cleavage (OPN-FL^mut^) on the EAE course* in vivo* in mice, since the functional* in vivo *role of the OPN cleavage and OPN fragments is far from being elucidated.

## 2. Materials and Methods

### 2.1. Production of Human and Murine Recombinant Proteins

Both human and mouse OPN cDNAs were purchased from imaGenes GmBH, Germany. The coding sequences lacking the signal sequence, of the OPN full length (OPN-FL) and the two thrombin-cleaved fragments (OPN-N and OPN-C), were amplified by PCR with specific oligonucleotides and cloned into pMB-SV5 vector [39] downstream from the immunoglobulin leader sequence. The thrombin-uncleavable OPN constructs (OPN-FL^mut^) was generated by mutating the mouse OPN sequence from AGGTCA coding for amino acids R_153_ and S_154_ to the AGCTTT coding for S_153_ and F_154_. This substitution has been described as yielding a thrombin cleavage resistant OPN [23]. In order to introduce the mutation, we performed site-directed mutagenesis using a mutagenic oligonucleotide specific for each sequence in combination with the reverse oligonucleotide mapping on the C-terminal end of the molecule. After amplification and restriction digestion, each mutated fragment was ligated with the wild-type upstream fragment obtained by restriction digestion to reconstitute the full length sequence. All these OPN constructs were subsequently amplified with specific oligonucleotides and cloned as six histidine- (6xHis-) tagged molecules into pUCOE vector [40]. For this cloning, we used a common forward oligonucleotide annealing on the Kozak sequence of the pMB-SV5 vector and a specific reverse oligonucleotide carrying 6xHis sequence. Recombinant OPN molecules were produced in Chinese Hamster Ovary Suspension Cells (CHOs; Invitrogen, Burlington, ON, Canada, USA). Cells were cultured in CHOS-SFMII medium (Invitrogen) and transfected with the pUCOE-OPN6xHis plasmids using FreeStyle MAX Reagent (Invitrogen) according to the manufacturer's instructions. To maintain stable transgene expression, transfected cells were cultured under selective pressure using 200 *μ*g/mL of hygromycin B (Invitrogen). The presence of each recombinant OPN construct in the cell supernatant was verified by western blotting using either an antibody directed against the His tag (Tetra-His Antibody, Qiagen, Valencia, CA, USA) or an anti-OPN antibody directed against an epitope located in the N- or C-terminal half of the molecule: SPP1 Polyclonal Antibody (Invitrogen) and Polyclonal Anti-Osteopontin Antibody (Millipore, Billerica, MA, USA), respectively. These antibodies were detected using the appropriate horseradish peroxidase-conjugated secondary antibody (Sigma-Aldrich, St. Louis, MO, USA). The reactive proteins were visualized by the ECL (Perkin Elmer, Waltham, MA, USA). All recombinant proteins were correctly recognized, and they displayed the expected size (i.e., 60 kDa for OPN-FL and OPN-FL^mut^, 35 kDa for OPN-N, and 25 kDa for OPN-C). Cells were grown at high density using CELLine*™* devices (BD Biosciences, San Diego, CA, USA) and collected twice a week. After centrifugation at 400 ×g for 10 minutes, cell supernatants were collected and each recombinant protein was purified on HIS Trap Excel Ni-Sepharose resin (GE Healthcare, Uppsala, Sweden), dialyzed overnight against PBS, and analyzed by western blotting and coomassie gel staining (Sigma-Aldrich).

### 2.2. Cells

Peripheral blood mononuclear cells (PBMCs) were separated from human blood samples obtained from healthy donors, who signed their written informed consent, by density gradient centrifugation using the Ficoll-Hypaque reagent (Lympholyte-H, Cedarlane Laboratories, Burlington, ON, Canada). The use of PBMCs was approved by the ethics committee of the “Azienda Ospedaliera Universitaria Maggiore della Carità” of Novara (Prot. 962/CE). CD4^+^ T cells and monocytes were negatively purified from PBMCs using the EasySep*™*Human CD4 Negative Selection Kit and EasySep Human CD14 Negative Selection Kit, respectively (Stem Cells Technologies, Vancouver, BC, USA). Cell purity was checked by immunophenotypic analyses and was higher than 95%. Peripheral blood lymphocytes (PBL) were obtained from PBMC after 2 h adhesion to remove monocytes. Cultures were performed in RPMI 1640 supplemented with 10% fetal bovine serum (FBS), and 100 U/mL penicillin, 100 *μ*g/mL streptomycin (Invitrogen). For interferon- (IFN-) *γ*, interleukin- (IL-) 17A, and IL-10 secretion and intracellular staining, 0.1 × 10^6^ CD4^+^ T cells were activated with anti-CD3 (1 *μ*g/mL, clone: OKT3) and anti-CD28 (2 *μ*g/mL, Ancell, Bayport, MN, USA) in the presence or absence of OPN-FL 1 *μ*g/mL, OPN-N, or OPN-C 0.5 *μ*g/mL for 5 days. For Tissue Inhibitor Metalloproteinase-1 (TIMP-1) and IL-6 secretion, 0.1 × 10^6^ monocytes were cultured in the presence or absence of OPN-FL 1 *μ*g/mL, OPN-N, or OPN-C 0.5 *μ*g/mL for 2 days. The effects of OPN-FL were compared to that of a commercial OPN-FL purchased from R&D System, and we obtained the same results.

Human umbilical vein endothelial cells (HUVECs) were isolated from human umbilical veins via trypsin treatment (1%) and cultured in M199 medium (Sigma-Aldrich) with the addition of 20% FCS (Invitrogen) and 100 U/mL penicillin, 100 *μ*g/mL streptomycin, 5 UI/mL heparin, 12 *μ*g/mL bovine brain extract, and 200 mM glutamine (HyClone Laboratories, South Logan, USA). Cells were grown to confluence in flasks and used at the 2nd–5th passage. The purity of the EC preparation was evaluated using morphologic criteria and positive immunofluorescence for factor VIII. Contamination with blood leukocytes was assessed via immunofluorescence with an anti-CD45 antibody. The use of HUVECs was approved by the institutional review board of the “Presidio Ospedaliero Martini” of Turin (Prot. 263-07/NF) that waived the need for consent; the data were analyzed anonymously and conducted in accordance with the Declaration of Helsinki.

### 2.3. ELISA

Concentrations of IL-17A, IFN-*γ*, IL-10, IL-6, and TIMP-1 were measured in culture supernatants by ELISA according to the instructions of the manufacturers (R&D System, Minneapolis, MN, USA; eBioscience San Diego, CA, USA and BioLegend, San Diego, CA, USA). Absorbance was detected with a microplate reader (Bio-Rad, Hercules, CA, USA), and the I-smart program was used to calculate the standard curve.

### 2.4. Intracellular Staining

CD4^+^ T cells (1 × 10^5^) were cultured for 5 days in round-bottomed 96-well plates in the presence of anti-CD3 (1 *μ*g/mL) plus anti-CD28 (1 *μ*g/mL) mAb and in the presence of recombinant OPN-FL 1 *μ*g/mL, OPN-N, or OPN-C 0.5 *μ*g/mL. After 5 days of culture, the cells were restimulated with phorbol 12-myristate 13-acetate (PMA, 50 ng/mL; Sigma-Aldrich) plus ionomycin (500 ng/mL; Sigma-Aldrich) for 5 hours in the presence of BFA (10 *μ*g/mL; Sigma-Aldrich). Then, cells were permeabilized, stained with a PE-conjugated anti-IL-10 mAb (Miltenyi Biotec GmbH, Bergisch Gladbach, Germany) and APC-conjugated anti-IL-17A mAb (eBioscience, San Diego, CA, USA), and analyzed by flow cytometry.

### 2.5. Cell Death Assay

Activation induced cell death (AICD) was evaluated on T cell lines obtained by activating PBMC with phytohemagglutinin (PHA, Sigma-Aldrich; 1 *μ*g/mL) and cultured in RPMI 1640 medium + 10% FBS + IL-2 (2 U/mL; Sigma-Aldrich) for 6 days. In the AICD assay, cells (5 × 10^4^/well) were cultured in wells coated with anti-CD3 mAb (OKT3, 1 *μ*g/mL) with RPMI + 5% FBS + 1 U/mL IL-2 in the presence or absence of OPN-FL (1 *μ*g/mL), OPN-N, or OPN-C (0.5 *μ*g/mL). Live cells were then counted in each well using the trypan blue exclusion test. Assays were performed in triplicate and results were expressed as relative cell survival % calculated as follows: (total live cell count in the assay well/total live cell count in the respective control well) × 100.

### 2.6. Cell Migration Assay

In the Boyden chamber (BD Biosciences) migration assay, resting PBL or HUVECs (5 × 10^4^ or 2 × 10^3^, resp.) were plated onto the apical side of 50 *μ*g/mL matrigel-coated filters (8.2 mm diameter and 0.3 *μ*m or 0.5 *μ*m pore size; Neuro Probe, Inc.; BIOMAP snc, Milan, Italy) in RPMI or M200 serum-free medium, with or without OPN-FL (10 *μ*g/mL), OPN-N (5 *μ*g/mL), or OPN-C (5 *μ*g/mL). Mediums containing 1 ng/mL RANTES (R&D System) or 10 ng/mL vascular endothelial growth factor (VEGF-*α*, R&D System) were placed in the basolateral chamber as a positive chemoattractant stimuli for PBL and HUVECs, respectively. The chamber was incubated at 37°C under 5% CO_2_. After 20 h, the cells on the apical side were wiped off with Q-tips. The cells on the bottom of the filter were stained with crystal violet, and all were counted (fourfold filter) with an inverted microscope (magnification 40x). Data are shown as percentages of the treated cells migration* versus* the control migration measured for untreated cells. Control migration is (mean ± SEM) 263 ± 45 cells for HUVECs (*n* = 5) and 155 ± 25 for lymphocytes (*n* = 5).

### 2.7. Cells Adhesion Assay

HUVECs were grown to confluence in 24-well plates in complete M200 medium (PromoCell GmbH, Heidelberg, Germany) and then treated or not with OPN-FL (10 *μ*g/mL), OPN-N (5 *μ*g/mL), or OPN-C (5 *μ*g/mL) for 30 min, washed with fresh medium twice, and incubated for 1 h with resting PBL (5 × 10^4^ cell/well). The 1 h incubation time was chosen to allow full sedimentation of the adhering cells, but similar results were obtained with a shorter incubation time (30 min). After incubation in the adhesion assay, nonadherent cells were removed by washing three times with M200. The center of each well was analyzed by fluorescence image analysis. Adherent cells were counted by the Image-Pro Plus Software for microimaging (Media Cybernetics, Bethesda, MD, version 5.0). Data are shown as percentages of the treated cells adhesion* versus* the control adhesion measured for untreated cells. This control adhesion was (mean ± SEM) 35 ± 4 cells per microscope field (*n* = 5).

### 2.8. Angiogenesis Assay

In the tube formation assay, HUVECs were cultured in M200 serum-free medium and seeded onto 48-well plates (2.5 × 10^4^/well) previously coated with 150 *μ*L of growth factor-reduced matrigel (BD Biosciences) in the presence of OPN-FL (10 *μ*g/mL), OPN-N (5 *μ*g/mL), OPN-C (5 *μ*g/mL), or control medium with VEGF-*α* (10 ng/mL, R&D System).

The morphology of the capillary-like structures formed by the HUVECs was analyzed after 6 h of culture using an inverted microscope (Leica Microsystem; magnification 10x) and was photographed with a digital camera (Leica Microsystem). Tube formation was analyzed and the number of tubes (with branching at both ends) was counted with an imaging system (Image-Pro Plus software for microimaging, Media Cybernetics, version 5.0, Bethesda, MD, USA). Tube formation was evaluated by counting the total number of tubes in three wells (*n* = 5) as previously described [41].

### 2.9. EAE Induction and OPN Treatment

Specific pathogen-free female C57BL/6 mice were purchased from Harlan (Harlan Laboratories, Indianapolis, IN, USA). The experimental protocol and animal handling were approved by CESAPO, the ethical committee of the University of Piemonte Orientale (Permit Number: 10/2013). To induce EAE, eight-week-old mice (*n* = 48) were immunized with 200 *μ*g of MOG_35–55_ peptide (Espikem, Firenze, Italy) emulsified in complete Freund adjuvant (Sigma-Aldrich) containing 4 mg/mL heat-killed* mycobacterium tuberculosis *(Difco laboratories, Detroit, MI, USA). On the day of MOG_35–55_ immunization and 48 h later, the mice were injected intraperitoneally (i.p.) with pertussis toxin (Sigma-Aldrich, 500 *μ*g in 0.1 mL of PBS). The mice were examined daily for clinical signs of EAE and scored as reported [42]. Twenty days after the remission, mice were divided into different experimental groups receiving daily injection of 5 *μ*g of different OPN variants (OPN-FL, OPN-N, OPN-C, or a mixture of OPN-C + OPN-N). Moreover, since OPN-FL is cleaved by thrombin* in vivo*, we also injected OPN-FL^mut^, lacking the thrombin cleavage site. Animal health was evaluated daily, throughout the duration of the experiment. Since the applied EAE protocol and the treatments with recombinant OPNs do not cause a long and intense suffering, we never administered an analgesic therapy. To prevent malnutrition in palsy mice, starting from EAE score 3.5 on, we put food and water directly into the cage, where they could easily reach. No unexpected death was recorded. Euthanasia was performed by cervical dislocation after a light inhalational anesthesia with isoflurane (2-chloro-2-(difluoromethoxy)-1,1,1-trifluoro-ethane) (ISOFLURANE VET FL VT, Merial Italia SpA, PD, Italy) using a precision out-of-circuit vaporizer (ISOTEC 4 series 1789, 2Biological Instruments SNC, VA, Italy) in a rodent induction chamber (2Biological Instruments SNC).

### 2.10. Data Analysis

The data are shown as the % of mean ± SEM. The statistical analyses were performed with GraphPad Prism 3.0 software (San Diego, CA, USA) using the Wilcoxon's signed rank test. The Friedman ANOVA test for repeated measures followed by Dunn's multiple comparison was used to analyze the daily clinical EAE score. *p* values <0.05 were considered significant.

## 3. Results

### 3.1. Production of Human and Murine Recombinant Proteins

**Figure 1:**
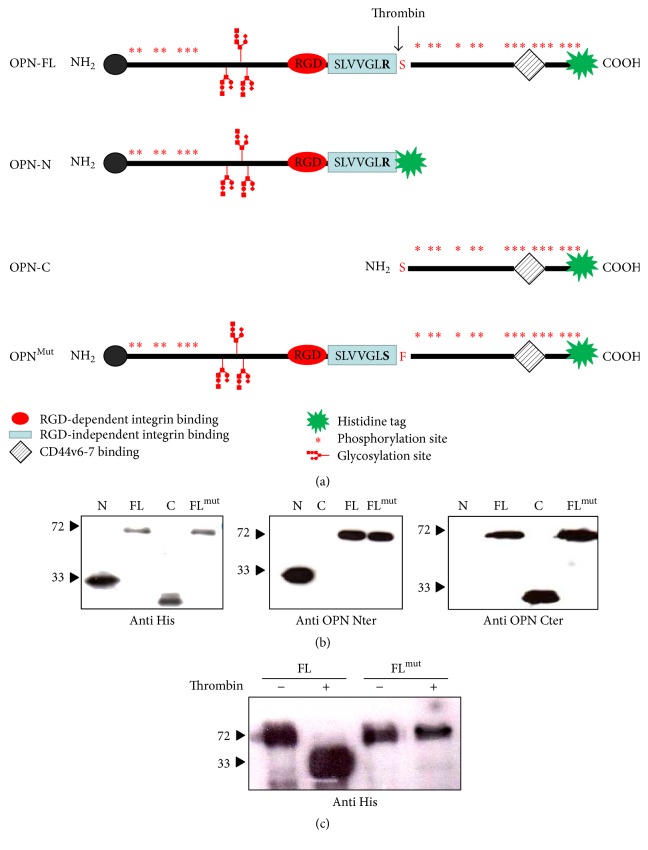
Recombinant OPN variants. (a) The figure depicts the recombinant OPN variants: OPN-FL (aa 17–314 human and aa 17–294 mouse), OPN-N including aa 17–168 (human) or 17–153 (mouse) of OPN; OPN-C including aa 169–314 (human) or 154–294 of OPN; mouse OPN-FL^mut^ carrying a mutated thrombin cleavage site (from R_153_-S_154_ to S_153_-F_154_). (b) Western blotting showing the recombinant proteins after purification probed with the anti-His-tag (left panel) or antibodies specific for the N (middle panel) or C-terminal portion (right panel). (c) OPN-FL but not OPN-FL^mut^ is cleaved by thrombin.

Both the human and murine leaderless OPN sequences, lacking the signal sequence, were cloned into pUCOE vector (OPN-FL). In order to assess the role of thrombin cleavage on OPN activity, we also cloned the following mouse and human OPN variants: OPN-N including aa 17–168 (human) or 17–153 (mouse) of OPN; OPN-C including aa 169–314 (human) or 154–294 of OPN; OPN-FL^mut^ carrying a mutated thrombin cleavage site (from R_153_-S_154_ to S_153_-F_154_) (Figure 1(a)) [23]. The cDNA coding for all these variants was cloned as fusion proteins with the 6xHis Tag and stably transfected into CHO cells. The presence of the recombinant proteins was verified in the culture supernatants by coomassie staining and by western blotting using antibodies designed against different epitopes of OPN or the His Tag (Figure 1(b)). All recombinant proteins displayed the expected sizes, that is, 60 kDa for OPN-FL and OPN-FL^mut^, 35 kDa for OPN-N, and 25 kDa for OPN-C, without presence of degradation products and/or contamination by other proteins. As expected, OPN-FL^mut^ was not cleaved by thrombin (Figure 1(c)).

### 3.2. Effects of OPN on Human Immune Cells In Vitro

**Figure 2:**
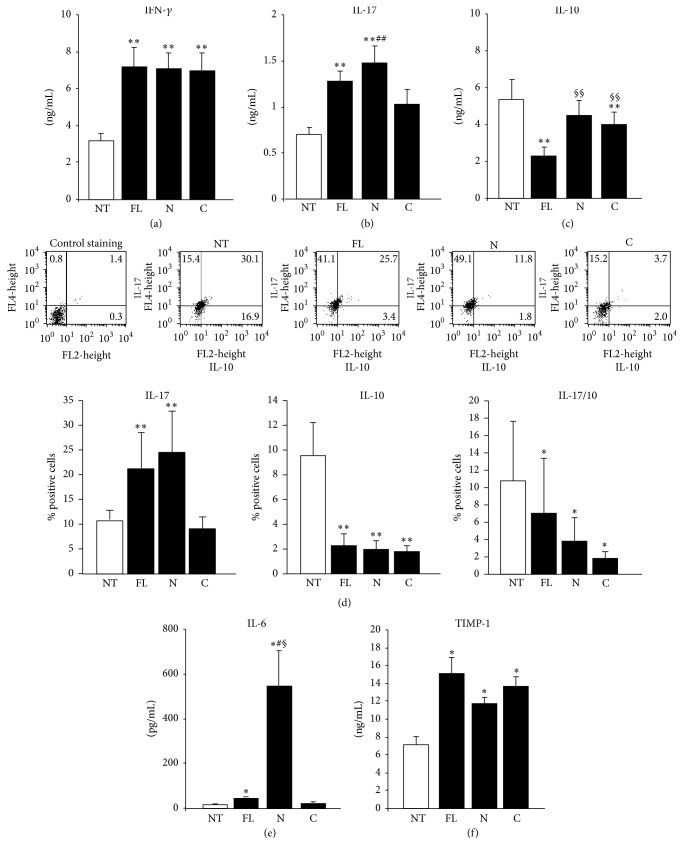
Effect of OPN fragments on cytokine secretion. (a) IFN-*γ*, (b) IL-17A, and (c) IL-10 protein evaluated in the culture supernatants from CD4^+^ T cells by ELISA or (d) by intracellular staining with anti-IL-17A and anti-IL-10 after 5 days of treatment with OPN variants. (e) IL-6 and (f) TIMP-1 protein secreted by monocytes after 2 days of treatment with OPN variants. Data are expressed as the mean ± SE from 6 independent experiments (^*∗*^ *p* < 0.05; ^*∗∗*^ *p* ≤ 0.01 versus the control; ^§^ *p* < 0.05; ^§§^ *p* ≤ 0.01 versus OPN-FL; ^#^ *p* < 0.05; ^##^ *p* ≤ 0.01 versus OPN-C; Wilcoxon's signed rank test).

We evaluated the effect of the human OPN-FL, OPN-N, and OPN-C on secretion of IFN-*γ*, IL-17, and IL-10 by T cells, since OPN is known to stimulate secretion of IFN-*γ* and IL-17 and to inhibit secretion of IL-10 [43, 44]. CD4^+^ T cells from healthy donors were activated by triggering CD3 and CD28 and cultured in the presence and absence of OPN-FL, OPN-N, and OPN-C for 5 days. Then, secretion of IL-17A, IFN-*γ*, and IL-10 was evaluated by ELISA in the culture supernatants. The results showed that all OPN preparations were similarly active in increasing secretion of IFN-*γ* (Figure 2(a)), whereas secretion of IL-17A was significantly increased by OPN-FL and OPN-N but not OPN-C (Figure 2(b)), and secretion of IL-10 was significantly decreased by OPN-FL and OPN-C but not OPN-N (Figure 2(c)). IL-10 and IL-17 expression was evaluated also by intracellular staining of cells restimulated for 5 h with PMA and ionomycin after the 5-day cultures. Cytofluorimetric analysis showed that the proportion of IL-17A single positive cells was significantly increased by OPN-FL and OPN-N but not OPN-C, whereas the proportions of IL-10 single positive cells and IL-17A/IL-10 dual positive cells were significantly decreased by all OPN preparations (Figure 2(d)).

We evaluated the effect of the human OPN-FL, OPN-N, and OPN-C on secretion of IL-6 and TIMP-1 in monocytes, since OPN is known to stimulate secretion of both molecules which play a key role in inflammation and several pathological conditions [45, 46]. Monocytes from healthy donors were cultured in the presence and absence of OPN-FL, OPN-N, and OPN-C for 2 days. Then, secretion of IL-6 and TIMP-1 was evaluated by ELISA in the culture supernatants. The results showed that secretion of IL-6 was induced mildly by OPN-FL and strikingly by OPN-N, whereas OPN-C had no significant effect (Figure 2(e)). By contrast, secretion of TIMP-1 was similarly induced by OPN-FL, OPN-N, and OPN-C (Figure 2(f)).

**Figure 3:**
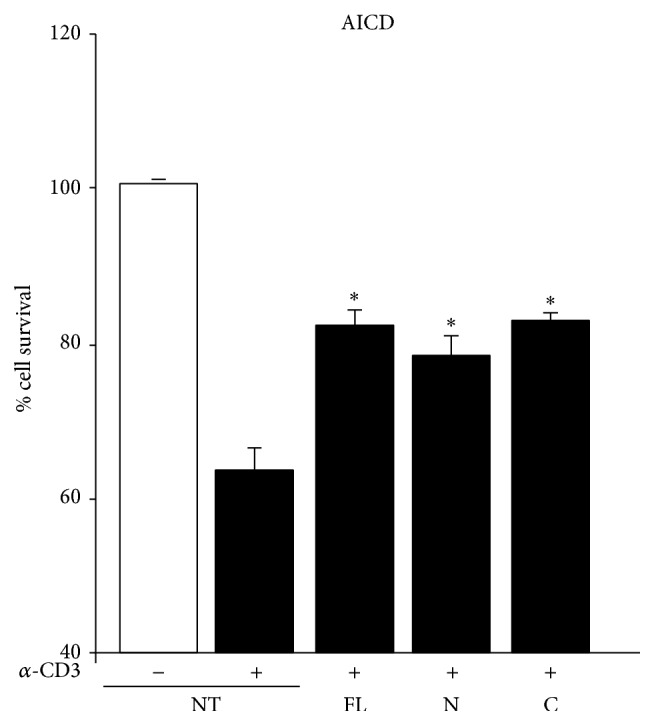
Effect of OPN variants on AICD of T cells. AICD was induced in PHA-derived T cell lines from healthy controls in the presence/absence of anti-CD3 and OPN variants. Results are expressed as relative cell survival % and are the mean ± SEM from 6 experiments. (^*∗*^ *p* < 0.05 versus the control; Wilcoxon's signed rank test).

We evaluated the effect of the human OPN-FL, OPN-N, and OPN-C in AICD of T cells, since OPN is known to inhibit T cell AICD, which is a key mechanism of peripheral tolerance [47]. PHA-activated T cells obtained from healthy donors were treated with anti-CD3 mAb to induce AICD in the presence and absence of OPN-FL, OPN-N, and OPN-C, and cell survival was evaluated after 16 h. The results showed that AICD was inhibited to the same extent by OPN-FL, OPN-N, and OPN-C (Figure 3).

**Figure 4:**
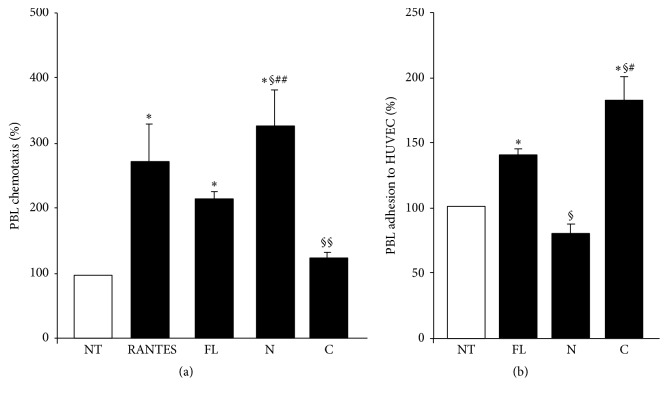
Effect of OPN variants on PBL migration and adhesion. (a) PBL were plated onto the apical side of matrigel-coated filters in 50 *μ*L of medium in the presence or absence of either 10 *μ*g/mL OPN-FL, 5 *μ*g/mL OPN-N, or OPN-C; RANTES (10 ng/mL) was loaded in the basolateral chamber as a positive control for migration. The cells that migrated to the bottom of the filters were stained using crystal violet and counted (5 fields for each triplicate filter) using an inverted microscope. (b) PBL were pretreated or not with OPN-FL, OPN-N, or OPN-C (10 *μ*g/mL) for 30 min, washed, and then incubated together for 1 h in the adhesion assay. Data are expressed as the mean ± SEM of the percentage of migration or adhesion versus the control obtained from untreated cells set at 100% from 5 independent experiments. (^*∗*^ *p* < 0.05; versus the control; ^§^ *p* < 0.05; ^§§^ *p* ≤ 0.01 versus OPN-FL; ^#^ *p* < 0.05; ^##^ *p* ≤ 0.01 versus OPN-C or OPN-N; Wilcoxon's signed rank test).

We evaluated the effect of the human OPN-FL, OPN-N, and OPN-C on lymphocyte migration and adhesion to vascular endothelial cells, since OPN is known to induce both cell adhesiveness and migration. In the migration experiments, PBL were seeded in the upper side of a Boyden chamber in serum-free medium; OPN-FL, OPN-N, and OPN-C and RANTES, used as positive controls for chemoattraction of lymphocytes, were loaded in the lower side of the Boyden chamber. The results showed that migration of lymphocytes was induced by OPN-FL and, to a greater extent, OPN-N, but not OPN-C (Figure 4(a)). In the adhesion experiments, HUVECs were treated with OPN-FL, OPN-N, and OPN-C for 30 min, washed, and then used in the adhesion assay with PBL. The results showed that adhesion was induced by both OPN-FL and, to a greater extent, OPN-C but not OPN-N (Figure 4(b)).

**Figure 5:**
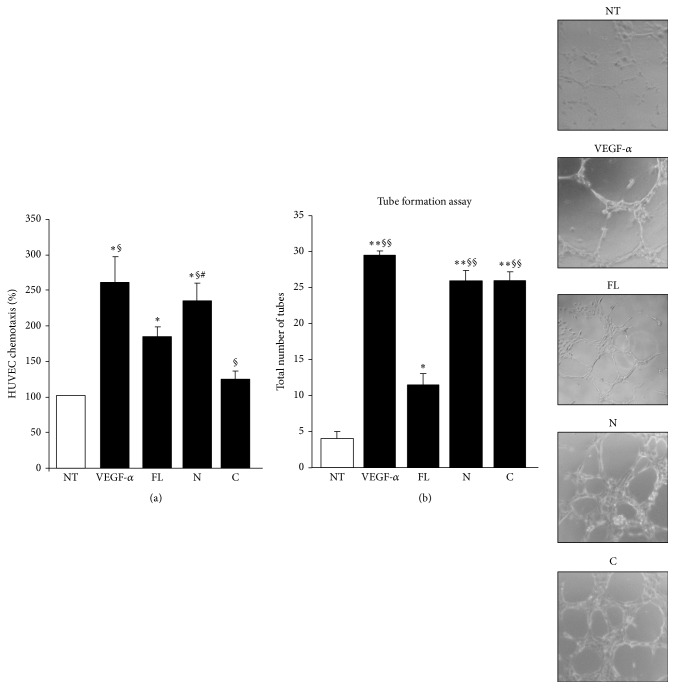
Effect of OPN variants on angiogenesis. (a) HUVECs were plated onto the apical side of matrigel-coated filters in 50 *μ*L of medium in the presence or absence of either 10 *μ*g/mL OPN-FL, 5 *μ*g/mL OPN-N, or OPN-C; VEGF-*α* (10 ng/mL) was loaded in the basolateral chamber as a positive control for migration. The cells that migrated to the bottom of the filters were stained using crystal violet and counted (5 fields for each triplicate filter) using an inverted microscope. Results are expressed as in Figure 4. (b) In the tube formation assay, HUVECs were plated in the presence and absence of OPN-FL (10 *μ*g/mL), OPN-N (5 *μ*g/mL), OPN-C (5 *μ*g/mL), or VEGF-*α* (10 ng/mL), as a control. The morphology of capillary-like structures formed by HUVECs was analyzed 6 h after culturing. Results are expressed as means ± SEM from 3 experiments (^*∗*^ *p* < 0.05; ^*∗∗*^ *p* ≤ 0.01 versus the control; ^§^ *p* < 0.05; ^§§^ *p* ≤ 0.01 versus OPN-FL; ^#^ *p* < 0.05; versus OPN-C; Wilcoxon's signed rank test). Right panels show a representative tubulogenesis experiment.

We evaluated the effect of the human OPN-FL, OPN-N, and OPN-C in angiogenesis* in vitro* by assessing migration and tube formation on HUVECs, since OPN is known to induce angiogenesis [48]. The migration assay was performed as with PBL using VEGF-*α* as a positive control. The results overlapped those obtained with PBL, since migration was induced by OPN-FL and, to a greater extent, OPN-N, but not OPN-C (Figure 5(a)). In the tubulogenesis assay, HUVECs were seeded onto growth factor-reduced matrigel in the presence or absence of OPN-FL, OPN-N, and OPN-C, and the morphology of capillary-like structures formed by HUVECs was analyzed after 6 h. The results showed that OPN-N and OPN-C induced high levels of tube formation, whereas OPN-FL had a mild, although significant, effect (Figure 5(b)).

### 3.3. Effect of OPN in Mouse EAE* In Vivo*

The first part of this work has been performed on human cells which are the therapeutic targets in human diseases. To assess whether the OPN forms exert different effects also* in vivo*, we moved to mouse EAE, a model of MS [32]. EAE is a T cell-mediated autoimmune disease characterized by perivascular CD4^+^ T cell and mononuclear cell infiltration, causing demyelination areas in the central nervous system, leading to progressive hind-limb paralysis.

Several works showed that, in the mouse, OPN displays similar effects than in human cells in terms of cytokine secretion [49], cell migration [50, 51], and angiogenesis [52]. Moreover, we performed pilot experiments using T cells and OPN variants from mice, which confirmed the pattern of OPN-FL, OPN-C, and OPN-N effects detected in the human model in terms of secretion of IFN-*γ*, IL-17, and IL-10, migration, and adhesion, which are key factors in EAE pathogenesis (data not shown).

**Figure 6:**
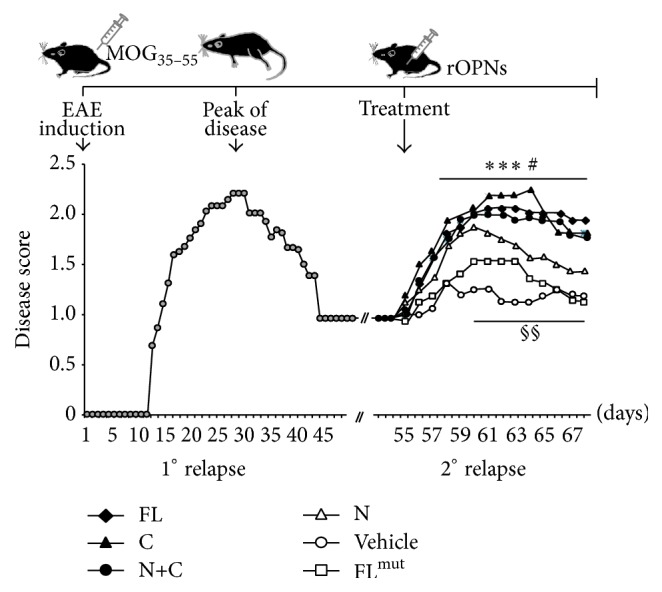
Effect of different forms of OPN on the EAE remission phase. The upper schema depicts the timing of the experiment: *n* = 48 mice were immunized at day 0 with MOG_35–55_ peptide to induce EAE. The mice were examined daily for clinical signs of EAE and scored as reported in the lower panel (grey circles). Twenty days after the remission, at day 55 after EAE induction, mice were randomized into different experimental groups and received daily injection of either OPN-FL (black diamonds), OPN-FL^mut^ (white squares), OPN-N (white triangles), OPN-C (black triangles), OPN-C + OPN-N (black circles), or vehicle (white circles). A nonparametric ANOVA test was used for clinical score comparisons (^*∗∗∗*^ *p* < 0.001 PBS versus OPN-FL, OPN-C, and OPN-N + OPN-C; ^#^ *p* < 0.05 PBS versus OPN-N; ^§§^ *p* < 0.01 OPN-C versus OPN-N and versus OPN-FL^mut^).

EAE can be induced by immunization with myelin proteins, such as proteolipid protein (PLP) or myelin basic protein (MBP) or myelin oligodendrocyte glycoprotein (MOG) in complete Freund's adjuvant (CFA). In C57BL/6 mice, the disease can be induced by immunization with the MOG immunodominant epitope corresponding to amino acids from 35 to 55 (MOG_35–55_). In this model, mice develop progressive paralysis (relapse) followed by a stable remission. However, administration of OPN in the remission phase induces a prompt relapse [34]. Therefore, we induced EAE in C57BL/6 mice and waited for the remission and, 20 days later, we started daily injections with the OPN variants and monitored relapse development. We compared the effect of the mouse OPN-FL, OPN-N, OPN-C, or OPN-C + OPN-N. Since OPN-FL* in vivo* is cleaved by thrombin, we also injected OPN-FL^mut^, lacking the thrombin cleavage site. The results showed that OPN-FL, OPN-C, and OPN-C + OPN-N induced a similar strong relapse of the disease (*p* < 0.001) (Figure 6), whereas OPN-N induced a mild relapse (*p* < 0.05) and OPN-FL^mut^ had no significant effect.

## 4. Discussion

In this study, we produced recombinant proteins corresponding to the human OPN-FL, OPN-N, and OPN-C. These proteins were produced in an eukaryotic system in order to ensure the posttranslational modifications influencing OPN functions, and they were used to investigate their individual activity on key players in the immune response, such as lymphocytes, monocytes, and endothelial cells. We also used the same approach to produce the mouse OPN-FL, OPN-N, and OPN-C, together with a thrombin-resistant form of OPN-FL in which the thrombin cleavage site was mutated (OPN-FL^mut^) and assessed their individual effect* in vivo* on mouse during EAE relapse.

In T cells, OPN cleavage seems not to influence the effect on IFN-*γ* secretion, which marks activation of Th1 cells since secretion of this cytokine is similarly costimulated by OPN-FL, OPN-N, and OPN-C. This suggests that production of IFN-*γ* is similarly costimulated by the triggering of either integrins or CD44, and no incremental effect is ascribable to the integrin cryptic site exposed in OPN-N. A similar reasoning may be applied to inhibition of AICD, which plays a key role in switching off the immune response and was similarly inhibited by OPN-FL, OPN-N, and OPN-C. This was not surprising, since both the RGD-dependent triggering of integrins and the triggering of CD44 v6-7 have been shown to protect cells from apoptosis [53, 54].

By contrast, secretion of IL-17 and IL-10, respectively, upmodulated and downmodulated by OPN-FL, differentially involves the two OPN fragments, since IL-17 upmodulation is mainly ascribable to OPN-N, whereas IL-10 downmodulation is mainly ascribable to OPN-C. These results are in line with reports showing that the OPN effects on IL-17 and IL-10 are selectively inhibited by anti-integrin and anti-CD44 antibodies, respectively [43].

The IL-17A secretion data were confirmed by intracytoplasmic expression of IL-17A. By contrast, the different effects of the OPN preparations on IL-10 secretion were not confirmed by intracytoplasmic staining of IL-10 since all OPNs similarly decreased the proportion of IL-10^+^ cells. Since cell staining was performed at day 5, whereas supernatant analysis evaluated the overall secretion during the whole culture time, it is possible that the supernatant differences were ascribable to initial phases of the culture. Another point is that all OPN preparations decreased the proportions of IL-10/IL-17A double positive cells, which suggest that they can work in pushing these cells toward a frankly proinflammatory Th17 effector function [55, 56].

A similar coordinated effect of OPN fragments can be envisaged on lymphocyte adhesion to endothelial cells and migration, which are two key steps of lymphocyte extravasation and homing into tissues. Our results, indeed, showed that both adhesion and migration are supported by OPN-FL, but adhesion is ascribable to OPN-C, whereas migration is ascribable to OPN-N, and each fragment displays its effect at higher levels than OPN-FL.

The experiment on HUVECs showed that also the OPN effect on migration of endothelial cells was ascribable to OPN-N and not to OPN-C. By contrast, both OPN-N and OPN-C displayed a strikingly higher effect than OPN-FL in inducing tubulogenesis, which is an* in vitro* assay of neoangiogenesis. These results are in line with the reports from Senger and colleagues [48] showing that VEGF induces OPN and *α*v*β*_3_ expression in endothelial cells and stimulates cleavage of OPN by thrombin and that the resulting OPN fragments are strongly chemotactic for endothelial cells and promote angiogenesis [57]. However, these authors used a mixture of the two OPN fragments obtained by* in vitro* thrombin-mediated cleavage of OPN-FL, and they could not distinguish the individual role of OPN-N and OPN-C. Moreover, other studies have shown that, in vascular endothelial cells, OPN enhances VEGF-*α* expression, which, in turn, mediates a positive feedback on OPN expression; the blocking of this feedback signal by anti-VEGF-*α* antibodies partially inhibits the OPN-induced HUVECs motility, proliferation, and tube formation [58].

In monocytes, the main finding was a marked putative effect of thrombin cleavage on IL-6 secretion, which was induced moderately by OPN-FL and strikingly by OPN-N, whereas OPN-C displayed no effect. This differential effect was detected on IL-6 but not on TIMP-1 whose secretion was similarly induced by all OPN preparations, and this suggests that OPN-N fine-tunes monocyte activation [32, 49, 59, 60].

Altogether our* in vitro* experiments show not only that the OPN effects on IL-17 and IL-6 secretion and cell migration are mainly ascribable to OPN-N, whereas those on IL-10 secretion and cell adhesion are mainly ascribable to OPN-C, but also that these effects are exerted at higher levels by the appropriate OPN fragment than by OPN-FL, which suggests that thrombin-mediated cleavage plays a key role in OPN activity. A striking gain of function was also detected in the tubulogenesis assay in which both OPN-N and OPN-C were much more active than OPN-FL. In the case of OPN-N, this gain of function may be ascribed to exposure of the cryptic integrin binding site that allows binding to integrins unbound by OPN-FL. By contrast, it is difficult to explain it for OPN-C, whose only known binding site is that for CD44, which is present also in OPN-FL. Besides the possibility that OPN-C exposes unknown cryptic binding site(s) for unknown ligand(s), the gain of function of OPN-C might be ascribed to removal of the N-terminal portion of OPN-FL, which may exert a partial steric or functional interference on CD44 triggering.

Also the* in vivo* experiments showed that thrombin-mediated cleavage of OPN plays a key role in OPN function, since OPN-FL was much more effective in inducing EAE relapses than OPN-FL^mut^, which is resistant to thrombin-mediated cleavage. Therefore, the effect of OPN-FL must be ascribed to the fragments produced by thrombin cleavage* in vivo*. Use of the recombinant OPN-C and OPN-N showed that the effect was ascribable to OPN-C, whereas OPN-N was active only weakly. The critical role of OPN-C was surprising, since the presence of the cryptic binding sites for *α*4*β*1 would instead draw the attention to OPN-N because *α*4*β*1 is involved in the homing of T cells into the central nervous system, and it is the target of the anti-MS drug natalizumab.

Substantial evidence indicates that OPN-FL plays a detrimental role in MS and EAE [32, 46, 61]. OPN-FL has been found to be the most abundantly expressed cytokine in the lesions of MS patients and EAE mice. Moreover, OPN deficient mice and mice injected with anti-OPN antibodies develop a mild EAE, whereas administration of OPN-FL triggers relapses in several EAE models [34]. Recently, proteomic analysis of MS lesions has unraveled a potential link between the coagulation cascade and MS pathology, which is supported by EAE data showing that administration of the thrombin inhibitor hirudin decreases clinical severity, demyelination, and Th1 and Th17 cytokines secretion [36, 38]. Intriguingly, thrombin activity is increased in the demyelinating lesions [37] where OPN is expressed at high levels. Our data confirm that thrombin-mediated cleavage of OPN plays a key role in MS relapse by exerting a dual effect. On the one hand, they may play a key role in the homing of autoreactive lymphocytes in the central nervous system lesions, since OPN-N increases production of IL-17 involved in breaking the blood brain barrier and stimulates lymphocyte migration, whereas OPN-C increases lymphocyte adhesion to vascular endothelial cells. On the other hand, they may support local inflammation, since OPN-N induces secretion of IL-6 in monocytes, OPN-C inhibits production of IL-10, and both increase secretion of IFN-*γ*.

## 5. Conclusion 

In conclusion, this study shows that the OPN fragments generated by thrombin exert distinct effects on cells involved in the immune and inflammatory response, and it suggests that drugs targeting each fragment may be used to fine-tune the wide effects of this cytokine.
